# Annotations

**Published:** 1891-08-29

**Authors:** 


### Annotations.

Our readers have probably seen elsewhere the story of Mrs. Aldred, who was tried at the recent Derbyshire Assizes for the murder of her infant child. She is the wife of a collier, who for years had treated her with a systematic brutality which is something incredible. Shortly before the occurrence which led to the trial just mentioned, stung by the remonstrances of the other women in the district, he shut his wife up in her room and offered her two alternatives, either to be stabbed with a knife which he displayed, or to be beaten. She chose the latter, and he stripped and deliberately beat her till she could not stand. A few days after a lodger missed a watch, and she was accused of taking it. Maddened by the treatment she had received, the miserable woman, to quote the words of the judge, decided to face God rather than her husband. She rushed from the house with the child in her arms, and soon after was discovered by some workmen in a neighbouring stream. A few yards distant was found the dead body of her child. On the bank was a pathetic letter to her "dear husband," saying that she and her baby had "gone out of the way." Technically she had murdered her child, but the jury acquitted her. Proceedings were taken against her husband, he was sentenced to the maximum penalty for his misdeeds—six months' imprisonment—but meanwhile he absconded. The woman is quite destitute, with two children dependent upon her and another expected. We have a few comments to make upon this revolting narrative. That the maximum penalty for such brutality should be six months' imprisonment is a disgrace to our criminal system. For years people have pointed out the gross injustice of punishing wife-beating with six months' imprisonment and petty larceny with two years' hard labour. It is usually a sensational case of this kind that brings about a change in the law, but unfortunately this is the silly season, and when Parliament meets again the story will have been forgotten. The saddest thing, however, is that such a thing should be possible in this country. Imprisonment is all very well, but it touches merely the symptoms and not the root of the evil. The incident presents a picture of what married life may mean in some classes of the community, which is absolutely heart-rending. There is one encouraging feature, and that is that the opinion of the district seems to have been strongly against the husband. Is it too much to hope that the spirit of chivalry may yet penetrate far enough to induce men like Aldred to reward the pathetic devotion of women to their "dear husbands" with something more than kicks and thrashings? For those who desire to render some help in this particular case we may say that contributions may be sent to the Rev. E. Muirhead Evans, Vicar of Ilkeston.

**Wife-Beating.**

The advent of county councils is likely to prove most inimicable to the voluntary system of hospital relief if we are to judge by the attitude recently taken up by the authorities in dealing with the rating of the Lock Hospital. This institution is one of the most useful and necessary of the metropolitan charities, and its administration has been markedly improved under the able management of Mr. Coote, the present Secretary. It is an odd way of encouraging the managers to improve their institutions for the local authorities to step in under such circumstances and increase the assessment for the poor rate from £150 to £1,668 without notice, *i.e.*, without affording the authorities an opportunity of making any objection or showing cause against the injustice of such a tax. It is unfortunately the law that overseers have the right to levy rates upon hospitals and kindred institutions at the present time. An experience of 25 years, and a careful consideration of the principles which govern taxation in this country, convinces us that the prospect of getting Parliament to exempt these institutions from the poor rate is remote, if not without hope. It must not be forgotten that the Legislature decided to tax Government buildings, as the result of a former agitation to procure exemption for hospitals and kindred charities. In these circumstances the only hope which the managers have is to cultivate cordial relations with the overseers who levy rates in the district where each institution is situated. It has been held by many overseers, and sanctioned by public opinion, that the only portion of a hospital which ought to be rated is that which is beneficially occupied. In other words, no rates can properly be levied upon any portion of such buildings except those which are occupied by the staff who receive remuneration for their services. We are afraid that the Lock Hospital authorities will not get any comfort from an appeal, because the overseers in this case have no doubt taken the value of the land and premises as the basis upon which their decision has been arrived at. Technically, therefore, they may be right in their decision though it is wholly opposed to public sentiment and justice. Nay, more, we are of opinion that the action of the overseers in this case is opposed to that of every other body of overseers in the country, and that if the authorities of the Lock Hospital take steps to bring the action of the local authorities under the notice of the ratepayers, and to organise (as they are bound to do in the best interests of their institution) an opposition to the re-election of those members who represent the district, in which the hospital is situated, they will find that any candidates they may nominate next election will be returned by a handsome majority. To rate a hospital exorbitantly is to make the charitable public who support it pay a double rate to the poor. Hospitals relieve the rates to a large extent not only by treating in-patients but by maintaining large out-patient departments. If the County Council insist upon the uttermost farthing they can legally wring from the hospital exchequer, they will find that in self defence the managers will have to curtail the amount of relief which they give, and so cause, much larger expenditure of the ratepayers' money by the Vestry Board who exhibit an unaccountable spirit of hostility to the most popular and beneficent of our voluntary charities. We would urge the Lock Hospital authorities to fight this question at the polls, because any other course must diminish the usefulness of the institution by crippling its funds and at the same time tend to injure the whole of the hospitals within the metropolis. They may rely upon it that if their case is properly represented the public will not support the action of the local authorities in this matter, and the sooner this is made plain to the Jacks-in-office who have gone out of their way to do a gross injustice to a great charity the better. The discourtesy and wantonness of the action of the local authorities towards the Lock Hospital is as unique as it is disgraceful. So much is this the case that we are of opinion that it proves the unfitness of those who made this rate for the public offices which they aspire to fill. The sooner the Board is changed the better it will be for the ratepayers of the Paddington district.

**The Rating of Hospitals.**

Shakespeare generalised too rashly when he stigmatised as cowards all those who "die many times before their deaths." Even brave men often look for death, though they may not fear it, when placed in unfamiliar circumstances. Put a sailor on a restive horse, put a huntsman in a tossing boat, and each, though valiant enough in his own sphere, will think his end at hand. Ignorance is the parent of many fears which fuller knowledge would dispel. Medical students, when they first begin their hospital practice, fancy themselves subject to every disease the lecturer describes, and sometimes fret themselves really ill with anxieties about their hearts and lungs. One could hardly imagine that these young men will, a few years later, walk fearless and unscathed through dangers they would hardly dare contemplate now. But fuller knowledge will teach them both what to dread and what to be indifferent to—above all, how best to secure such protection as they can, and leave the rest to God. A few weeks ago there was a great outcry against the inhabitants of Mull because they refused shelter to a pedler, whose worst fault was that he had helped to place in his coffin a man who had died of fever. Refused admittance to one house after another, he struggled on until, worn out, he fell and died by the wayside. Yet the Highlanders are neither cowardly nor cruel, but they may be excused for hesitating before they received into their houses what they regarded as the bringer of certain death. Had they known that a milder measure of quarantine would have sufficed, they would have bidden the pedler wash himself and let him sleep in an outhouse at the least. In the middle-ages men were brave, if they were nothing else; yet the mediæval notion of stamping out a disease was the drastic measure of killing those who were suffering from it lest it should infect themselves; and then they would let the putrefying corpses lie about and do the very mischief they so dreaded. Courage and kindness are both born of wisdom, and the spread of knowledge is the best cure for both the folly of rashness and the folly of fear.

**Dying Daily.**

